# Production of radiometals in liquid targets

**DOI:** 10.1186/s41181-019-0088-x

**Published:** 2020-01-10

**Authors:** Sergio J. C. do Carmo, Peter J. H. Scott, Francisco Alves

**Affiliations:** 1 ICNAS — Produção, Pólo das Ciências da Saúde, University of Coimbra, Azinhaga de Santa Comba, 3000-548 Coimbra, Portugal; 2 Department of Radiology, University of Michigan, Ann Arbor, MI 48109 USA; 3 ICNAS – Institute for Nuclear Sciences Applied to Health, Pólo das Ciências da Saúde, University of Coimbra, Azinhaga de Santa Comba, 3000-548 Coimbra, Portugal; 4 IPC - Instituto Politécnico de Coimbra, Coimbra Health School, 3046-854 Coimbra, Portugal

**Keywords:** Cyclotron, Radioisotope production, Liquid target, Radiometals

## Abstract

Over the last several years, the use of radiometals has gained increasing relevance in supporting the continuous development of new, complementary and more specific biological targeting agents. Radiopharmaceuticals labelled with radiometals from elements such as Tc, Zr, Y, Ga and Cu received increasing attention as they find application in both diagnostic SPECT and PET imaging techniques and radiotherapeutic purposes. Such interest stems from the wide variety of radionuclides available with distinct and complementary nuclear decay characteristics to choose from with unequalled specificity, but can also be explained by growing demand in targeted radionuclide therapy. As a result, as routine supply of these radiometals becomes mandatory, studies describing their production processes have expanded rapidly. Although most radiometals are traditionally provided by the irradiation of solid targets in specialized cyclotrons, recently developed techniques for producing radiometals through the irradiation of liquid targets have received growing attention due to compatibility with commonly available small medical cyclotrons, promising characteristics and encouraging results. Irradiating liquid targets to produce radiometals appears as a fast, reliable, convenient and cost-efficient alternative to the conventional solid target techniques, characterized by complex and time-consuming pre- and post-irradiation target handling. Production of radiometals in liquid targets incorporated to complete manufacturing processes for daily routine is already recognized as a viable alternative and complementary supply methodology to existing solid target based infrastructures to satisfy growing clinical demands. For instance, several sites already use the approach to produce ^68^Ga-radiopharmaceuticals for clinical use. This review article covers the production of common radiometals with clinical potential through the irradiation liquid targets. A comparison with the traditional solid target irradiation methods is presented when relevant.

## Introduction

Over the last several years, interest in biomedically useful molecular probes labelled with radionuclides other than common positron emitters like ^11^C, ^13^N, ^15^O and ^18^F has increased significantly (Reichert et al. 1999; McQuade et al. 2003). Although the development of new tracers based on common radionuclides such as ^11^C and ^18^F will continue to play a significant role in future developments in Positron Emission Tomography (PET) radiochemistry, there is a growing demand and great potential for developing more specific targeted agents for which these more traditional position emitters are inadequate (McQuade et al. 2003; Nedrow et al. 2011). For instance, some biological mechanisms are simply too slow when compared to the short half-life of ^18^F (110 min) and corresponding imaging tools and therefore require radionuclides with much longer half-life to enable to quantify radioactivity, tracer kinetics and biological uptake beyond few hours after administration. As other radionuclides are required to enlarge the spectrum of biological processes that can be studied (and treated) (Pagani et al. 1997), radiolabelling with non-conventional radionuclides has become mandatory to support and answer the continuous development of novel molecular probes for Single Photon Emission Computed Tomography (SPECT) and PET diagnostic imaging techniques and also for radiotherapeutic applications (Cutler et al. 2013). In that context, the use of radioactive metal ions (radiometals) has to be considered (Reichert et al. 1999), as they offer an abundant, varied and complementary range of radionuclides to choose from, according to their nuclear decay characteristics regarding half-life, decay mode and energy and branching ratios (see Table 1 for common examples). Such diversity is of significant importance as it makes possible to select with unequalled specificity the nuclear properties most suitable to match the biological characteristics for each molecular probe, either for diagnostic or therapeutic purposes (Cutler et al. 2013). Radiolabelling of molecules with radiometals is based on the incorporation of the radiometal into the intended biologically active targeting molecule though a linking agent, denominated as chelator. This intermediary is a bifunctional compound as it simultaneously presents a complex extracting the radiometal from its solution and guaranteeing its stable immobilization, and a group assuring the bond to the vector molecule (Price and Orvig 2014). While the molecular probe is determined by the intended target receptor, the choice of chelator depends on the pharmacokinetic requirements of the pharmaceutical and the radiometal (Cutler et al. 2013; Price and Orvig 2014; Wadas et al. 2010; Fani et al. 2011).

**Table 1:** Emerging radiometals and their nuclear properties

Radiometal	Half-life		β- end-point	Average	Maximum	Average	Electron	Characteristic
			energy	β^−^ energy	β^+^ energy	β^+^ energy	Capture	γ-rays
			(keV)	(keV)	(keV)	(keV)	(%)	(keV)
diagnostic radionuclides								
^43^Sc	3,89	h			1198,8 (70,9%)			372,8 (23%)
					862,4 (17,2%)			
^44^Sc	3,97	h			1474 (94,3%)	632	5,7	1157 (99%)
								1499 (0,91%)
^45^Ti	184,8	min			1040,4 (84,82%)			720,2 (0,154%)
^61^Cu	3,33	h			559,5 (2,5%)	239	38,3%	283 (12,0%)
					932,5 (5,4%)	398,9		908,6 (1,12%)
					1148,1 (2,1%)	493,8		1185 (3,6%)
					1215,5 (51,6%)	523,8		
^63^Zn	38,3	min			1382,2 (4,96%)	599,5		669,9 (8,19%)
					1674,3 (7,00%)	732,0		962,0 (6,50%)
					2344,2 (80,3%)	1041,9		1412,1 (0,74%)
^64^Cu	12,7	h	579 (38,5%)		653 (17,52%)	278	43,9	1346 (0,475%)
^68^Ga	67,8	min			821,7 (1,2%)	352,6	11,1	1077 (3,24%)
					1899 (88,9%)	836,0		1883 (0,142%)
^86^Y	14,74	h			1220,6 (11,9%)	535		627,7 (32,6%)
					1545,2 (5,6%)	681	66	777,4%(22,4)
					1988,3 (3,6)	883		1076,6 (83%)
					3141,3 (2,0%)	1437		1153,0 (30,5%)
^89^Zr	78,4	h			902 (22,8%)	395,7	77%	908,97 (99,0%)
^94m^Tc	51,9	min			570 (0,43%)	198,5		871,09 (94,04%)
					917 (0,91%)	404,8		993,2 (2,21%)
					1446 (0,41%)	639,6		1521,9 (4,48%)
					2439 (67,2%)	1094,4		1868,79 (5,49%)
therapeutic radionuclides								
^47^Sc	3349	d	441,4 (68,5%)	142,8				159,4 (68,5%)
			600,8 (31,5%)	204,2				
^67^Cu	2,58	d	392,4 (57%)					91,27 (7,0%)
			483,7 (22%)					93,3 (16,1%)
			577 (20%)					184,58 (48,7%)
^90^Y	2668	d	518 (0,02%)	163,7				
			2278,7 (99,98%)	926,7				
^161^Tb	6,88	d	461,3 (26%)					25,65 (23,2%)
			518,5 (66%)					48,92 (17,0%)
			567,45 (10%)					74,57 (10,2%)
^166^Ho	26,83	h	1774,3 (48,7%)					80,6 (6.71%)
			1854,9 (50,0%)					1379.4 (0.93%)
^177^Lu	6,64	d	177,0 (11,64%)	47,66				112,95 (6,2%)
			385,4 (9,1%)	111,7				208,37 (10,38%)
			498,3 (79,3%)	149,4				
^211^At	7,22	h	5.87 MeV α-particles (41,8%)					
^225^Ac	10,0	d	5.79 MeV α-particles (18.1%)					
			5.83 MeV α-particles (50,7%)					

The complementary variety of nuclear properties is sufficiently appealing by itself to justify the growing interest in radiometals. However, their increasing popularity has also been enhanced by the fact that the many of them present radioisotopes suitable for imaging and therapy, leading to *theranostics*. In such procedures, the same molecular probe can be labelled with either a diagnostic radionuclide for imaging or a therapeutic radioisotope, using a suitable diagnostic radionuclide to non-invasively confirm the presence and distribution of the intended target so as to confirm eligibility for the corresponding image-guided personalized molecular therapeutic treatment. The selection, planning and optimization of a given radiotherapeutic strategy based on the information obtained from imaging with the diagnostic radiopharmaceutical leads to a more selective and thus efficient treatment, while minimizing radiation dose to healthy tissues. On the other hand, the improved results from personalized clinical therapy can stimulate the discovery of new biological targets and support future research and development (R & D) efforts. For instance, a therapeutic radionuclide is chosen so that the radiation emitted is deposited with high probability within the intended target in the malignancy: the decay mode and radiation range must therefore match the target size, texture, location and geometry. The irradiation of tissue volumes with multicellular, cellular, and subcellular dimensions are therefore typically performed with β particles, α particles, and Auger electrons, respectively (Zalutsky 2011; Muller et al. 2017). Most currently used therapeutic radiometals are listed in Table 1. Molecular theranostics represent a powerful platform to which radiometals are essential, and a breakthrough opening a new era in nuclear medicine that has been motivated significantly by positive clinical results (Lee and Li 2011). In this regard, the growing demand for radiopharmaceuticals labelled with radiometals has therefore also been actively driven by increasing interest in theranostic radionuclide pairs for combined imaging and therapy, i.e. radiometals with similar properties (starting with indispensable comparable half-lives), ideally from the same element, and exhibiting diagnostic and therapeutic properties. The theranostic match pairs ^68^Ga/^177^Lu, ^68^Ga/^90^Y, ^64^Cu/^67^Cu, ^86^Y/^90^Y, ^43,44^Sc/^47^Sc and ^149,152^Tb/^149,155,161^Tb, among others, have been used in clinical applications to date (Cutler et al. 2013; Qaim 2019; Qaim et al. 2018). In certain cases, a single radionuclide can present both properties as is the case for ^64^Cu (both β^+^ and β- decays) and ^149^Tb (both β^+^ and α decays).

However, although the increased interest in radiometal isotopes has been consensually identified over the past years and their major role in future radiopharmaceutical developments is also predicted, clinical application of radiopharmaceuticals labelled with radiometals remain moderate (Notni and Wester 2018). Besides, it is also rather difficult to explain why a given radiometal with no particularly outstanding feature has been most successfully used over others by simply comparing its decay characteristics. As discussed by Notni et al. (Notni and Wester 2018), this contradictory scenario might be explained by the fact that two fundamental conditions have to be simultaneously fulfilled in order to witness the breakthrough of a given radiolabelled molecular probe; namely the availability of the required radionuclide and its clinical application. The importance of availability indeed surpasses most characteristic considerations and is therefore the most fundamental attribute of a radionuclide, regardless of the suitability of its nuclear decay characteristics. ^64^Cu is a perfect example of that paradox since its use in clinical applications is still limited despite its long established recognition as an emerging radionuclide by the imaging and radiotherapy communities (Blower et al. 1996; Smith 2004). Indeed, ^64^Cu provides a low-energy positron comparable to ^18^F, which represents a particularly significant advantage in preclinical studies where spatial resolution in small-animal PET scanners is relevant, and it decays emitting both β^+^ and β- particles with an appealingly long 12,7 h half-life for certain applications. Besides, the chemistry of copper is restricted to two principal oxidation states (I and II), while the coordination chemistry of copper is diverse and well established in the design of radiopharmaceuticals (Reichert et al. 1999; Wadas et al. 2010; Anderson et al. 2003). Nevertheless, the potential of such interesting features have not yet been fully exploited in both preclinical and clinical applications because of the high price and insufficient commercial availability of ^64^Cu. For instance, the enriched ^64^Ni necessary for the ^64^Ni(p,n)^64^Cu nuclear reaction is costly (around 30 k€ per gram). On the other hand, a perfect example of this contradictory popularity is ^68^Ga which, despite its high positron energy and unappealing short half-life for distribution (Table 1), stands out as the most successful radiometal in recent years thanks to its much more convenient and cost-effective availability through the use of ^68^Ge/^68^Ga generators. Table 2 presents radiometals available through generators with well-established clinical interest. Although this subject is of particular relevance in the context of radiometals (Roesch and Knapp 2011), the theme also goes beyond the scope of this review and is therefore not addressed in detail. However, by presenting the nuclear decay characteristics of generator-produced radioisotopes, which are not superior to other emerging radiometals, Table 2 illustrates that availability plays a major role in the implementation of a specific radionuclide. ^68^Ga is also a perfect example to illustrate the vital importance of clinical applications since, despite such convenient availability, the success of ^68^Ga-based radiopharmaceuticals was only triggered in 2001 by the clinical relevance of [^68^Ga]Ga-DOTA-TOC, and only reached peak popularity with the approval of a second clinically successful ^68^Ga-based radiopharmaceutical (Notni and Wester 2018), [^68^Ga]Ga-PSMA-11. Finally, despite such success, even more widespread use of ^68^Ga has been limited because of the relatively low activity levels delivered by ^68^Ge/^68^Ga generators (≤3.7 GBq) and the associated long down-time between elutions (≤4 h); at least until a few years ago with the introduction of the cyclotron-production of ^68^Ga in liquid targets as explained later. The same discussion can be applied to therapeutic nuclides. For example, ^90^Y was the uncontested leader up to 2000 because it was found to be effective and have low toxicity (Notni and Wester 2018). However, interest in ^177^Lu is increasing and demand can be expected to slowly catch up with the demands for ^90^Y. For example, [^177^Lu]Lu-DOTA-TATE and [^177^Lu]Lu-PSMA-617, both recently acquired by Novartis, are approved and in advanced clinical trials, respectively. Among many other suggested therapeutic radionuclides, also ^161^Tb arouses interest to as it produces similar or even improved clinical results in comparison to ^177^Lu, while ^225^Ac and ^213^Bi are attracting attention for alpha therapy (Müller et al. 2014; Grünberg et al. 2014).

**Table 2:** Generator-produced radiometals with clinical potential

Generator	Parent	Daughter nuclide								References
	nuclide	Half-life	β^−^ end-point	Average	Maximum	Average	Electron	Typical activity	Generator	
			energy	β^−^ energy	β^+^ energy	β^+^ energy	Capture	per elution	longevity	
	Half-life		(keV)	(keV)	(keV)	(keV)	(%)	(GBq)		
^44^Ti/^44^Sc	60,6 y	3,97 h			1474 (94,3%)	632	5,7	0,18	1–2 years	(Pruszynski et al. 2010; Filosofov et al. 2010)
^62^Zn/^62^Cu	9,26 h	9,74 min			2910(98%)		2	1,2	1 day	(Anderson et al. 2003; Haynes et al. 2000)
^68^Ge/^68^Ga	271 d	1,14 h			821,7 (1,2%)	352,6	11,1	0,370-1,85	1–2 years	(Fani et al. 2008)
					1899 (88,9%)	836,0				
^82^Sr/^82^Rb	25,6 d	1,26 min			2604 (13,1%)	1168	4,35	200	1–6 months	(Phillips et al. 2000)
					3381 (81,2%)	1535				
^90^Sr/^90^Y	28,6 y	64,1 h	518 (0,02%)	163,7				18,5	tens of years	(Chakravarty et al. 2008)
			2279 (99,9%)	926,7						
^188^W/^188^Re	69,4 d	17,0 h	1487 (1,6%)	527,8				20	10 months	(Dasha and Knapp Jr 2015)
			1965 (25,6%)	728,9						
			2120 (71,1%)	795,4						

The latter considerations concerning the importance of both availability and clinical application aim at illustrating the vital importance of discussing production methods. Indeed, supply issues for a given radioisotope might limit severely or even preclude its use, regardless of the potential for clinical purposes. The International Atomic Energy Agency (IAEA) precisely reported that the use of some radionuclides for clinical applications have been limited, despite the firmly increasing interest observed over the last years, because the support of the radiopharmaceutical industry lacks large scale and economically attractive production processes (Cyclotron produced radionuclides 2016). As a result, this work intends to present the improvements reported in recent years concerning the production of radiometals in liquid targets in cyclotrons; as recent results (IAEA-TECDOC-1863 2019) suggest its potential as a complementary technological breakthrough in an effort to provide large scale production routes to radiometals that are suitable for daily routine use; at a time that the number of available and dedicated medical cyclotrons has undergone significant growth.

### The production of radiometals

In order to respond to the increasing demands for the vast portfolio of emerging radiometals, possible production routes for each radioisotope of interest have been considered, studied and compared through the years. Detailed reviews and technical reports on the subject are therefore available, addressing both reactor- and cyclotron-based production routes (IAEA 2010; IAEA 2009; Qaim 2011). However, as these latter routes are dated with respect to recent developments on the production of radiometals in liquid targets, such reviews describe mostly applications of the solid target technique. Taking into account this default, this article reviews the technical aspects involved in the production of radiometals using cyclotrons equipped with liquid targets, and also introduces the technique as an opportunity to upgrade existing resources in PET centers equipped with dedicated cyclotrons. Indeed, although some exceptions require higher particle energies, a large number of relevant radiometals can be successfully produced making use of already existing medical cyclotrons presenting proton energies up to 20 MeV and commonly dedicated to the production of ^18^F and/or ^11^C. Emerging radiometals that can be produced using biomedical cyclotrons are presented in Table 3. These particular radionuclides were chosen due to their relevance in the scope of the present work.

**Table 3:** Solubility of different substances of interest for the production of emerging radiometals, in water at 1 atm pressure and 25 °C (Handbook of chemistry and physics 2005)

Intended	Target	Salt	Solubility of the salt in water @ 25 °C (g/100 g H_2_O)	Maximum concentration of target material in water @ 25 °C (g/100 g H_2_O)
Radiometal	Element			
^43^Sc / ^44^Sc	Ca	Ca (NO_3_)_2_	144	35
^61^Cu/^68^Ga	Zn	Zn (NO_3_)_2_	120	41
		ZnCl_2_	408	196
^61^Cu/^64^Cu	Ni	Ni (NO_3_)_2_	99,2	32
^86^Y	Sr	Sr (NO_3_)_2_	80	33
^89^Zr	Y	Y (NO_3_)_3_	149	48
		YCl_3_	75	34
^94m^Tc	Mo	(NH_4_)_6_Mo_7_O_24_	43	4,4
^63^Zn	Cu	Cu (NO_3_)_2_	145	49

Although cyclotron targets can be found for all three states of matter, gases and liquid targets are usually used for low atomic weight target elements because of the chemical form found in nature (e.g. production of short half-life positron emitters such as ^11^C or ^18^F). On the contrary, solid targets are mostly dedicated to the irradiation of medium/high atomic weight target nuclides, especially metals, as they are naturally available in solid form (e.g. production of radionuclides with longer half-lives). The solid target technique is also superior, even mandatory, when large activities of radionuclides with longer half-lives (including a large number of radiometals) are required, as it enables simultaneously the use of larger beam currents (improved solid target assemblies stand up to hundreds of μA, while recently developed liquid targets only stand up to 140 μA), higher densities of target nuclides while also providing superior production yields. For instance, some studies report irradiations accumulating tens of thousands of μAh (Meinken et al. 2005; Fassbender et al. 2004).

However, although standard solid target solutions are nowadays available from multiple manufacturers, the irradiation of solid targets also usually comes with the challenges of high cost and tremendous operational complexity. First of all, facilities equipped for the use and transport of irradiated solid targets are scarce and it is rather difficult and expensive to implement adapted solutions on existing infrastructures. Modifications aimed at handling and transport of solid targets can represent a cost-prohibitive and insuperable obstacle. Besides, the problem worsens for self-shielded cyclotrons as these are not prepared to allocate a solid target station. Besides facility and cyclotron considerations, handling solid targets also suffers from complex and time-consuming practical difficulties before and after irradiation. Several reports have shared detailed and complex preparatory procedures including the preparation of the backing support (a process than can last tens of hours (Adam-Rebeles et al. 2013)) and sophisticated electrodeposition processes (Adam-Rebeles et al. 2013; Ometakova et al. 2012). The latter requires both thermal resistance tests and surface quality analysis prior irradiation (Rajec et al. 2010; Kakavand et al. 2010) as these affect the production yield. The irradiation of such solid targets is also of concern as impinging currents mean that difficulties during irradiation might lead to the loss of enriched target material through evaporation (McCarthy et al. 1997a). The post-irradiation handling, transport and dissolution of the irradiated solid target (and its inevitably activated backing support) are also particularly important issues as these are time-consuming and especially delicate in terms of radiation protection. Such lengthy post-irradiation procedures are not prohibitive when considering production routes requiring post-irradiation cooling time to allow radio-impurities with shorter half-lives to decay, but they are a particularly important issue when radioisotopic impurities with longer half-lives are also produced and impact the product quality with time thus limiting its shelf life (e.g. the residual presence of ^66^Ga and ^67^Ga when producing ^68^Ga (Alves et al. 2017a)). The dissolution of the solid target thick layer is also followed by recovery and recycling procedures for the large amount of expensive enriched material, resulting thus in loss of material not inferior to 5% (McCarthy et al. 1997a; Stoll et al. 2002; Obata et al. 2003). These obstacles mean that the irradiation of solid targets is therefore unlikely to be used for the production of short-lived radioisotopes. Finally, one can also anticipate increased difficulties when consecutive irradiations are required.

As a result, distinct and complementary production techniques with faster post-irradiation procedures are inevitably required for radioisotopes with shorter half-lives. Simpler and faster production, purification and synthesis procedures are also fundamental to achieve efficient production routines for daily use (Alves et al. 2018). In this context, liquid targets present several practical advantages while simultaneously avoiding several of the practical difficulties associated with the use of solid targets. Liquid targets are rapidly and remotely transported with ease, and deliver the produced radioisotopes in a more convenient chemical state. Besides convenience, liquid targets avoid the major investment in a solid target system and the production of a single batch of liquid target solution is enough to be used in several irradiations, which can be consecutive when necessary. The use of liquid targets to produce non-conventional radioisotopes was first suggested in 1973 by Lindner et al. (Linder et al. 1973; Schimmel et al. 1979), and later by Cuninghame et al. (Cuninghame et al. 1976), for the production of ^123^I using flowing liquid targets. This technique was also used in the following years by Hutter et al. (Hutter et al. 1992) and Galiano et al. (Galiano and Tilbury 1998) for the production of ^77^Br, and by Vogg et al. (Vogg et al. 2004) to produce ^86^Y. It was only in more recent years, with the increasing clinical interest in emerging radiometals and the significant growing number of biomedical cyclotrons simultaneously, that the production of radiometals in liquid targets has actually become more of a necessity than simply a possibility. Reflecting this, several radiometals of interest have been produced in biomedical cyclotrons through the irradiation of liquid targets very recently. Such promising results in such short time, resulting in convenient and efficient manufacturing processes with potential to enhance the availability in more radioisotopes predict an aggressive expansion of the technique in the near future. For instance, the IAEA already included and pointed out the development of the liquid target technique in their latest report on the cyclotron production of ^68^Ga (IAEA-TECDOC-1863 2019).

The production of radiometals in liquid targets is based in the dissolution of the target material in a strong acid and then diluting the resulting metal-salt in acid solution (typically in 0,01 to 1 M range) for loading into the liquid target. Salt-based target solutions inevitably present lower densities of target material, and thus lower production yields when compared to the corresponding solid targets. However, recent results from several authors demonstrate that the technique provides sufficient activities for daily clinical use, with improved convenience because significantly reduced post-irradiation processing (and associated decay) partially compensates for the lower activity levels produced. Table 3 presents the solubility of target materials of interest in water, keeping in mind that the dissolution in acid solutions is even more favourable. Contradictory, although the use of fewer target nuclides was previously pointed out as an inconvenience when long half-life isotopes and/or large activities are required, it actually represents a significant practical advantage in liquid targets for the production of radioisotopes with half-lives less than a few hours: the possibility of choosing the amount of enriched target material to dissolve enables scaling adequately the concentration of the target solution according to the production need, minimizing production costs. In fact, even for solid targets, the use of thinner layers is usual in order to reduce the operating costs; not making therefore full use of the much larger production yields available anyway (Avila-Rodriguez et al. 2007). Even so, savings in the amount of target material is limited in the case of solid targets since the requested target area is defined by the beam profile.

Cyclotron targets (either gas, liquid or solids targets) are usually composed of a single element (isotopically enriched if necessary), or a single molecule containing the target element of interest (e.g. H_2_^18^O), and thus involve very few chemical agents. Carriers and additives to dictate the output chemical form, or to enhance the production yield and quality, are exceptions. However, the irradiation of metal-salt based solutions to produce radiometals introduces novel and more complex circumstances as these are mainly composed of distinct non-target elements, with the intended target material being only a dissolved part of the overall irradiated target. As a result, additional chemical interactions need to be considered when more complex mixtures are irradiated. The irradiation of metal-salt based liquid targets for the production of radiometals therefore presents particular characteristics over conventional liquid targets, which users need to be aware of: firstly, the diluting solution is acidic and demands great care in the choice of materials involved. Such consideration naturally concerns the target insert and the entrance target foil, but also all other materials in contact with the irradiated solution in the overall production process, i.e. valves, fittings, tubing and transfer lines used in both the target assembly and the synthesis system. Usable materials have to avoid corrosion in order to maintain their mechanical strength and also to guarantee inertness during the production process. For instance, aluminium was found to be inadequate (Chakravarty et al. 2013), while commonly used Havar® is problematic due to its iron content. For instance, iron shares chemical properties with gallium and thus critically affects the complexation of ^68^Ga during downstream radiochemistry (Hoehr et al. 2017). This latter case is also a perfect example of how the choice of materials also depends on the intended radiometal and its production route. Another concern also arising from the use of acid target solutions is accelerated target foil degradation when compared to the irradiation of conventional liquid targets. Several groups have reported observing weakened target foils and increased risk of failure, ranging from discoloration to material loss (Alves et al. 2017a; Hoehr et al. 2017; Zacchia et al. 2017). They commonly attribute such enhanced degradation to the foil/salt interface in contact with the acid solution while under bombardment, but any comparison is rather difficult as these observations resulted from very different productions conditions (distinct foil materials, beam energies and currents, target pressures, salts, acids and pH). Zacchia et al. analysed irradiated target foils presenting severe degradation when used for the production of radiometals (Zacchia et al. 2017). These were much more profoundly affected by temperature when compared to those irradiated for routine ^18^F production, suggesting that heat is not the only responsible agent. They also performed bench tests intending the reproduction of the target conditions during bombardment, suggesting that the formation of reactive radical species (e.g. ^•^NO_2_) may result in significant foil degradation.. On the other hand, Alves et al. observed no limiting degradation of the foil with superior beam currents up to 70 μA, nor unexpected failures occurring, as long as more frequent substitution of the foil is guaranteed (Alves et al. 2017a). Moreover, while the irradiation of pure water presents equal rates for the formation and the recombination of radicals, resulting thus in a steady-state with minimal quantities of gas produced, the presence of metal-salt in the water modifies this equilibrium (Zacchia et al. 2017) and the possibility of formation of species using H^•^ or OH^•^ radicals reduces their recombination and promotes radiolysis. Some of the resulting species formed are gases that cause higher pressures in the target and thus limit the current that can be applied to the target. Indeed, several groups have observed continuous rises in pressure inside liquid targets from beginning of irradiation with constant target currents, sometimes so significant that the irradiation has to be interrupted after only a few minutes (Hoehr et al. 2017; Zacchia et al. 2017; Pandey et al. 2014b). On the contrary, such effects were not observed by other authors (Alves et al. 2017a; Alves et al. 2017b; do Carmo et al. 2017) and these discrepancies will likely be explainable as the communities experience with production of radiometals in liquid targets increases in coming years. Additional studies also report production difficulties caused by precipitation of salts inside the target (Zacchia et al. 2017; Oehlke et al. 2015). Thus it is important to rinse such targets with dilute acid solutions, and not water as is common for ^18^F targets. Distinct and contradictory irradiation conditions have been reported by different groups, but all agree on the importance of the composition of the target solution in the production yield. A systematic study on the subject was performed by Pandey et al. and showed that the rate of gas evolution depends on both the cation and anion solutes and also on their concentration (Pandey et al. 2014a). In this study into the optimization of the liquid target for the production of ^89^Zr, they demonstrated that YCl_3_ produced much larger amounts of gaseous side products when compared to Y (NO_3_)_3_, whereas NaCl led to much lower gas formation than NaNO_3_. They also verified that increasing the YCl_3_ concentration resulted in an increase in the formation of gas, whereas no change was observed when increasing the concentration of Y (NO_3_)_3_. The study concluded that chloride salts present increasing gas evolution for heavier cations, while nitrate salts result in growing gas formation for lighter cations (in Group 1–3 metals). These authors also tried to reduce gas evolution through additives including nitric acid and ethanol; the addition of 1 M nitric acid reduced gas evolution by about 50% for almost all of the salts considered, with simultaneous elimination of salt precipitation, whereas ethanol resulted in huge formation of precipitates in the target and was thus abandoned. The benefit from the addition of nitric acid was attributed to a diminution of the interaction of hydroxyl radicals with metal cations. For constant anion concentrations, nitrate ions showed similar gas evolution for the cations considered, while chloride ions showed increasing gas evolution as the cation mass increases. Third and finally, the irradiation of such liquid target solutions inevitably lead to the production of large amounts of ^13^N (more than 50 GBq for larger beam currents) and also of some ^18^F (up to 1 GBq for long irradiations), due the ^16^O(p,α)^13^N and the ^18^O(p,n)^18^F nuclear reactions on water, with this latter becoming relevant when the irradiation time increases (as necessary for the production of radioisotopes of longer half-lives such as ^64^Cu). Other distinct radionuclides are also produced, depending on the acid used to dissolve the target material. For example, ^11^C is produced through the ^14^N(p,α)^11^C reaction when using nitric acid. As a result, the assessment of the radionuclide produced through γ-spectrometry is inevitably limited prior separation during the first hour after EOB, while the activity of ^13^N is still considerable, independent of the production route.

Results from recent studies regarding the production of radiometals in liquid targets are summarized in Table 4. The Table also presents characteristics from the solid target technique for comparable production conditions. These studies confirm the expected superior production yields achieved with the solid target technique, but also show that the production yields at saturation obtained with the irradiation of liquid targets are sufficient to satisfy clinical applications. Indeed, most of the reported produced activities are still limited because from preliminary irradiations, i.e. of reduced length and with limited target currents, but there is also thus plenty of room for improvement to scale up the developed methods to achieve more useful activities. Besides, since the production yield depends naturally on the concentration of target material dissolved, a metric of interest to investigate refers to MBq.cm^3^/(μA.g) at saturation or MBq.cm^3^/(μAh.g) for normalization, and to assist in planning irradiations (Alves et al. 2017b).

**Table 4:** Production characteristics for the radiometals obtained in biomedical cyclotrons through the irradiation of liquid targets for both the liquid target and solid target techniques

Radionuclide	Nuclear reaction	Liquid target technique					Solid target technique		References
		Proton	Concentration		Produced	Production	Concentration	Production	
		energy	of target nuclide	Irradiation	Activities	yield	of target nuclide	yield	
		(MeV)	(mg/ml*)*		(MBq)	(MBq/μA _*@ sat*_)	(g/cm3)	(MBq/μA _*@ sat*_)	
^44^Sc	^*nat*^Ca(p,x)^44^Sc	12	180	60 min @ 7,8 μA	6,3	1,94-4,6			(Hoehr et al. 2014)
		16					1,55	179	(Valdovinos et al. 2015)
	^*44*^Ca(p,n)^44^Sc	11					1,15	400	(Krajewski et al. 2013)
^61^Cu	^*nat*^Zn(p,x)^61^Cu	16,9	250	45 min @ 25 μA	300	83			(do Carmo et al. 2017)
				3 h @ 70 μA	2270				(do Carmo et al. 2018)
	^*nat*^Ni(p,x)^61^Cu	–	494	50-120 min @ 20 μA	–	–			(Engelbrecht et al. 2013a)
	^*nat*^Zn(p,x)^61^Cu	22 → 12					7,13	2811	(Rowshanfarzad et al. 2006)
		11,4						100	(Asad et al. 2016)
^64^Cu	^64^Ni(p,n)^64^Cu	16,9	25	300 min @ 70 μA	5000	300			(Alves et al. 2017b)
		–	15	120 min @ 10 μA	90	87			(Engelbrecht et al. 2013b)
		11,4					8,91	5883	(Avila-Rodriguez et al. 2007)
		15,5						3642	(McCarthy et al. 1997b)
^63^Zn	^63^Cu(p,n)^63^Zn	14	232-321	60 min @ 20 μA	2000–3060	151–309			(DeGrado et al. 2014)
	^*nat*^Cu(p,x)^63^Zn	13,5 → 11,4					8,96	1688	(Guerra Gomez et al. 2012)
^68^Ga	^*nat*^Zn(p,n)^68^Ga	12	307	60 min @ 7 μA	480	141			(Oehlke et al. 2015)
	^68^Zn(p,n)^68^Ga	14	116	30 min @ 20 μA	2250	430			(Pandey et al. 2014b)
		16,1-12,9	33	50 min @ 45 μA	6000	330			(Alves et al. 2017a; Alves et al. 2017b)
		12	115	32 min @ 46 μA	4200	320			(Riga et al. 2018)
		15					7,14	8872	(Sadeghi et al. 2009)
^86^Y	^*nat*^Sr(p,x)^86^Y	12	196	60 min @ 4,6 μA	7,4	31			(Oehlke et al. 2015)
	^86^Sr(p,n)^86^Y	–	137	60-120 min @ 10–15 μA	500–1000	743			(Ralis et al. 2010)
		14,2 → 8,4					2,1	1253	(Lukic et al. 2009)
		16						1820	(Schmitz 2011)
		14,5 → 10,4					3,97	3848	(Yoo et al. 2005)
^89^Zr	^*nat*^Y(p,n)^89^Zr	14	177,8-244,5	60–120 min @ 20–40 μA	81–349	500			(Pandey et al. 2014a) (Pandey et al. 2016)
		12	204,0	60 min @ 7,3 μA	32	360			(Oehlke et al. 2015)
		15					4,47	6970	(Holland et al. 2009)
^94m^Tc	^*nat*^Mo(p,x)^94m^Tc	13	177-541	60 min @ 5 μA	12–110	16–40			(Hoehr et al. 2012)
		13 → 7					6,5	2600	(Roesch et al. 1994)
		12,1						602	(Szajek et al. 2003)
		14,7						1964	(Bigott et al. 2001)

### ^68^Ga

Jensen and Clark (Jensen and Clark 2011) introduced the use of a liquid target for the production of the particularly relevant ^68^Ga as an appealing alternative to ^68^Ge/^68^Ga generators. The ^68^Ge/^68^Ga generator presents undeniably several advantages as it is a convenient source of ^68^Ga for daily use, lasting for months and enabling independence from ^68^Ga production centres. It can also be used for the preparation of kit-based radiopharmaceuticals as it holds a manufacturing authorization. However, the latter solution also present several limitations such as the high cost of the generator (which is actually still increasing significantly), poor availability (18 month lead times have been reported), limited lifetime (~ 9 months) with decreasing eluted activities over time, the limited activity of ^68^Ga per elution (~ 1–2 GBq), the down-time between elutions (up to 4 h), and the t risk of breakthrough of the long half-life parent ^68^Ge. The down-time between elutions is a particularly relevant limitation as the demands for ^68^Ga are expected to continue to increase rapidly, making a single generator possibly insufficient for routine clinical supply. Besides the immediate interest due to its well established (and still growing) clinical relevance, ^68^Ga is a perfect example of the utility of the irradiation of salt-based liquid targets because its short half-life is much less compatible with the use of the solid target technique for routine manufacturing. The preliminary results presented by Jensen and Clark, making use of the well-known ^68^Zn(p,n)^68^Ga nuclear reaction on ^68^ZnCl_2_ salts, were promising and helped the authors to immediately identify and stress the potential of the technique for local radiopharmaceutical production. They reported residual presence of the radioisotopic impurity ^67^Ga. Indeed, although the presence of ^67^Ga can be massively reduced by keeping the energy of the impinging protons near and below the 12,0 MeV threshold of the ^68^Zn(p,2n)^67^Ga nuclear reaction, the ^67^Zn(p,n)^67^Ga reaction remains unavoidable, particularly when 100% enriched ^68^Zn is not available. Residual presence of ^66^Ga has also been reported for the same reasons, i.e. due to ^66^Zn(p,n) Ga reaction on the residual ^66^Zn content (Alves et al. 2017a; Alves et al. 2017b). Pandey et al. (Zacchia et al. 2017) and Hoehr et al. (Hoehr et al. 2017; Hoehr 2014) also produced latter ^68^Ga through the irradiation of liquid targets. They faced and outstripped some experimental difficulties identified previously by Jensen and Clark but still reported limited activities of ^68^Ga at EOB, namely 0,96 and 0,445 GBq at EOB respectively. A significant improvement in the production of ^68^Ga in liquid targets was latter reported by Alves et al. (Alves et al. 2017a; Alves et al. 2017b) with routine production of 5–6 GBq of ^68^Ga per batch, with strong perspectives for improvement up to about 25 GBq by increasing the concentration of ^68^Zn for instance. Not only such enhanced activities, with fewer enriched material used, definitively represent a major improvement when compared to the activities delivered by generators but this group also pioneered successful labelling of DOTA- and HBEB- peptides under Good Manufacturing Practice (GMP) by using ^68^Ga produced through the irradiation of liquid targets. Decay-corrected purification and labelling yields of about 80 and 65% respectively were reported, resulting in complete production process of ^68^Ga-labelled radiopharmaceuticals in agreement with European Pharmacopeia in less than one hour after EOB. More recently, Riga et. al*.* (Riga et al. 2018) also produced significant amounts (~ 4,2 GBq of ^68^Ga) while degrading the incident proton energy down to 12 MeV in order to improve the radionuclidic purity.

### ^64^Cu

Simultaneously with the successful production of ^68^Ga in liquid targets, Alves et al. also developed a similar technique to produce ^64^Cu (Alves et al. 2017b). As already referred, the interest in ^64^Cu is relevant because it presents particularly relevant characteristics, among which one has to point out its low-energy positron, similar to ^18^F (Williams et al. 2005). However, its much longer half-life is also compatible with longer irradiations and the production of about 5 GBq of ^64^Cu after 5–7 h of irradiation has been reported (Alves et al. 2017b). Once again, the authors pointed out that there is much room for improvement and that the technique enables the production of about 20–25 GBq of ^64^Cu by simply scaling up the concentration of ^64^Ni. In this particular case, the possibility of choosing the concentration of ^64^Ni depending on the production needs is particularly relevant as the required enriched ^64^Ni is very expensive. Since ^64^Cu is the radioisotope of copper produced with a longer half-life, the purity of the ^64^Cu produced improves with time after EOB and also with increasing irradiation length because the production of the other radioisotopic impurities reach saturation long before ^64^Cu (Alves et al. 2017b). The ^64^Cu produced was purified with decay-corrected yields of about 70–80% and used for several labelling purposes (e.g. [^64^Cu]Cu-ATSM). ^64^Cu was also produced by irradiating ^64^Ni(NO_3_)_2_ by Engelbrecht et al. (Engelbrecht et al. 2013b). The authors were able to produce about 90 MBq of ^64^Cu after 2 h of irradiation with 10 μA and using 45 mg of enriched ^64^Ni. However, such results correspond to a reduced production yield at saturation when compared to Alves et al. (Alves et al. 2017b), only partially explained by the distinct amounts of ^64^Ni and proton energies (Table 4).

### ^61^Cu

As previously referred, implementation of the emerging ^64^Cu has still been rather limited because of two main reasons: i) its limited availability and ii) the production costs involved due to the cost-prohibitive ^64^Ni. Because of its recent enhanced availability thanks to the salt-based liquid target technique, significantly growing demand for ^64^Cu is predicted. However, routine production of ^64^Cu therefore represents significantly increased demands for ^64^Ni, with a consequent large investment to be made in all initial stages of development and optimization of new ^64^Cu labelled radiopharmaceuticals. In an effort to attenuate such an obstacle, ^61^Cu has been considered as a complementary and useful alternative to ^64^Cu because it is faster and much less expensive to produce and naturally shares any chemistry platforms developed for ^64^Cu (do Carmo et al. 2017). ^61^Cu can be used as a substitute for ^64^Cu whenever possible, thus reducing significantly the costs of products development. ^61^Cu has a convenient 3,3 h half-life and a positron decay branch superior to that of ^64^Cu (61,5% vs. 17,5%) with no emission of β^−^ (Table 1). Its 1,2 MeV average positron energy is far superior to that from several other radioisotopes, such as ^68^Ga for instance (Williams et al. 2005). ^61^Cu was produced with convenience though the irradiation of inexpensive natural zinc thanks to the ^64^Zn(p,α)^61^Cu reaction (do Carmo et al. 2017). The reduced cost of the target material enables the use of much larger quantities of zinc in the target solution and almost saturated solution with 250 mg/ml were used to produce 0,3 GBq of ^61^Cu in only 45 min long preliminary irradiations using small volume targets (do Carmo et al. 2017). The purification process ensures the elimination of all contaminants other than radioisotopes of copper (mainly ^66^Ga, ^67^Ga and ^68^Ga), thus guaranteeing that the ^61^Cu produced only contains the radioisotopic contaminant ^64^Cu produced through the ^67^Zn(p,α)^64^Cu and ^68^Zn(p,nα)^64^Cu reactions. The radionuclidic purity of ^61^Cu was determined to be ~ 97,5% at EOB, a value that decreases with the length of the irradiation but also remains above 95% several hours after EOB. The target system was subsequently improved and the irradiation scaled up so that production of ^61^Cu increased up to about 2,3 GBq (do Carmo et al. 2018). This is a quantity that certainly reinforces the stated potential of ^61^Cu as a viable and useful solution. Detectable quantities of ^61^Cu were also produced by Engelbrecht et al. (Engelbrecht et al. 2013a), also aiming to use it as a substitute for ^64^Cu in developmental work to avoid the use of expensive enriched ^64^Ni. However, in this latter work, ^61^Cu was produced via the ^61^Ni(p,n)^61^Cu reaction by irradiating solutions with natural nickel dissolved as Ni (NO_3_)_2_.

### ^89^Zr

As for the cases of ^61^Cu and ^64^Cu, the interest in positron emitters with longer half-lives, making shipping and distribution possible, fostered further development in salt-based liquid targets for the production of radiometals as ^86^Y, ^44^Sc and ^89^Zr. As referred earlier when describing the importance of the cation and anion species, their concentration and the dissolving media in the behaviour of salt-based solutions under bombardment is important. Pandey et al. dissolved YCl_3_ and Y (NO_3_)_3_ salts in several distinct solutions for studying the production of ^89^Zr (Table 4) (Zacchia et al. 2017). The improved process they developed enabled the production of 349 MBq of ^89^Zr by irradiating a target solution composed of a 2 M solution of yttrium nitrate dissolved in 1,25 M nitric acid with 40 μA of 14 MeV protons for 2 h (Pandey et al. 2016). Oehlke et al. also irradiated solutions of dissolved yttrium nitrate with similar concentrations to produce ^89^Zr (Oehlke et al. 2015). Their lower 7,3 μA target current and proton energy of 12 MeV protons explained the lower activities produced and the reduced production yield at saturation, showing agreement with the results from Pandey et al. *(*Pandey et al. 2016*)* (Table 4). In all cases, the proton energy was chosen to avoid the production of the undesirable long-lived ^88^Zr (t_1/2_ = 83,4 days).

### ^44^Sc

Regarding ^44^Sc, it has been available through the ^44^Ti/^44^Sc generator (Table 2). Such generators would in theory represent a convenient choice since it is a perfect example of a secular equilibrium, with a very long-lived parent (63 years) and a short-lived daughter (3,97 h), and the eluted solution would present activities similar to the activity loaded into the generator thousands of times over many years. However, in practice, the half-life of the parent ^44^Ti (63 years) is too problematic since the production of sufficiently large quantities is very difficult and therefore cost-prohibitive, and the increasing breakthrough over many years becomes unacceptable. However, the interest in ^44^Sc still remains unaffected as it forms an appealing theranostic pair with ^47^Sc, a promising low-energy β^−^ emitter. Moreover, as Sc^3+^ presents chemical properties and stability similar to Y^3+^ and Lu^3+^ to form DOTA conjugates - even improved when compared to Ga^3+^ − ^44^Sc-labeled radiopharmaceuticals are highly attractive as distribution becomes possible. Hoehr et al. dissolved ^*nat*^Ca (NO_3_)_2_ in water, resulting in solutions with concentration of ^*nat*^Ca up to 180 mg/cm^3^ (Table 4) (Hoehr et al. 2014). Their preliminary short irradiations at 20 μA enabled the production of 28 MBq of ^44^Sc and they estimated the production of 88 MBq of ^44^Sc by simply scaling up the irradiation time to 4 h. It is also possible to predict the production of about 4,2 GBq of ^44^Sc with the production process described by using enriched ^44^Ca instead of ^*nat*^Ca (2,09% of ^44^Ca).

### ^86^Y

Despite its decays is accompanied by the emission of undesired high energy γ-rays, the relevance of ^86^Y as an emerging radiometal for diagnostic PET imaging increased considerably because it forms a suitable matching theranostic pair with the more and more relevant therapeutic nuclide ^90^Y, and they also present similar half-lives (Table 1). As the demands for ^90^Y are steadily increasing, production by irradiation of a liquid target therefore also finds application in satisfying the consequent growing needs in ^86^Y. Oehlke et al. demonstrated that the production of ^86^Y is possible by irradiating ^*nat*^Sr (NO_3_)_2_ (Oehlke et al. 2015). They produced 7 MBq of ^86^Y after one hour of irradiation with only 4,6 μA on target. Much larger quantities of ^86^Y were recovered by Ralis et al. also by irradiating aqueous solutions with strontium nitrate but by using enriched ^86^Sr (Ralis et al. 2010). They reported the production of up to 1 GBq of ^86^Y after 2 h irradiation at 15 μA.

### ^63^Zn

Following their work on the production of ^68^Ga and ^89^Zr in liquid targets, DeGrado et al. used salt-based liquid-targets to produce ^63^Zn (DeGrado et al. 2014). Enriched ^63^Cu was used to dissolve ^63^Cu(NO_3_)_2_ salts in nitric acid and to prevent the production of the long-lived ^65^Zn via ^65^Cu. The authors reported the production of 2–3 GBq of ^63^Zn after 60 min irradiations at 20 μA. Such quantities were used to administrate ^63^Zn-zinc-citrate for PET imaging in mice (DeGrado et al. 2014), and later in human participants (DeGrado et al. 2016).

### ^94m^Tc

Finally, ^94m^Tc was also considered of interest as a surrogate of the relevant ^99m^Tc for PET imaging (Hoehr et al. 2012). Hoehr et al. obtained target solutions containing natural molybdenum by dissolving ammonium heptamolybdate (NH_4_)Mo_7_O_24_ in water with hydrogen peroxide (Hoehr et al. 2012). The production of up to 110 MBq was confirmed after 60 min irradiation with 5 μA on target, making possible the production of about 1,1 GBq of ^94m^Tc with the present production process by using enriched ^94^Mo.

Table 4 summarizes the studies concerning radiometal production from liquid targets in the last decade. Table 4 also presents and confirms the superior yields achieved with the solid target technique, for each radioisotope considered, for comparison. As the different works concerning the production of radiometals in liquid targets stress benefits of this technological platform in comparison to the more conventional solid target or generator approaches, more manufacturing processes involving the production of radiometals through the irradiation of liquid targets are expected in a near future, including further adaptation of the procedures described herein. For instance, it is quite straight forward to produce ^66^Ga or ^67^Ga adapting the platform developed for ^68^Ga, while ^43^Sc can be produced by modifying the process created for the production ^44^Sc. ^43^Sc has almost identical half-life to ^44^Sc, while presenting lower-energy positron and much less energetic and fewer undesirable γ-rays (Table 1).

It seems relevant to refer at this point that accelerator manufacturers are nowadays commercializing liquid targets and also creating machines with variable energy or modifying fixed-energy biomedical cyclotrons because some production routes require lower energies for optimized production with the highest purity (IBA 2018), e.g. the referred ^68^Ga or ^89^Zr radiometals. The possibility of customizing directly the optimal incident energy for each production route instead of using a degrader, avoiding thus target currents limitations due to undesirable power dissipation and beam shape alteration, is expected to bring major improvements in terms of current on target and therefore increased production yields at the optimal energy level. Such innovation, which places such cyclotrons as an optimal compromise between fixed-energy cyclotrons delivering protons in the 16–20 MeV energy range and low-energy machines with production yields too small in most cases to be considered for daily routine productions, is particularly relevant for liquid targets as minimized power dissipation in the target window will certainly attenuate limitations due to target pressures and foil degradation. Among all targets, it is expectable that this technological breakthrough will find direct application in stimulating and benefiting the implementation production processes of radiometals in liquid targets.

## Conclusion

The increasing interest in radiometals for diagnosis and therapeutic purposes will continue to stimulate and foster the development of novel and more efficient production processes to guarantee their routine supply in the global nuclear medicine market. In that context, the introduction of the production of radiometals through the irradiation of liquid targets for cyclotron-produced radioisotopes appears as a viable and convenient complementary alternative to the solid target technique and to the convenient generators systems. These latter invariably present the same practical problem: generators possess either a parent nuclide with an adequate long half-life, but which is synonymous of limited availability, limited and decreasing eluted activity, high cost and permanent and increasing risk of unacceptable breakthrough, or a parent nuclide of with relatively short half-life thus limiting its longevity. On the contrary, the irradiation of salt-based liquid targets can easily and rapidly be incorporated into existing facilities while simultaneously reducing production costs. Such enhanced characteristics and practical convenience present potential to improve the accessibility to several radiometals of interest; a vital step to transform some up to now occasional production procedures into the fully routine processes required to satisfy the steadily growing demands. In recent past years, the irradiation of liquid targets already resulted in the production of several radiometals of interest; namely ^44^Sc, ^61^Cu, ^63^Zn, ^64^Cu, ^68^Ga, ^86^Y, ^89^Zr and ^94m^Tc. These studies reported distinct procedures ranging from preliminary irradiations to fully automated production processes leading to the manufacturing of radiopharmaceuticals. In all cases, the potential of the salt-based liquid target technique was recognized as a viable and complementary alternative to be considered in the portfolio of cyclotron-based techniques for the production of radioisotopes; with more radiometals of interest therefore expected to be produced through the irradiation of liquid targets in upcoming years, as the demands for more specific target agents will steadily continue to growth.

## Data Availability

Please contact author for data requests.

## Abbreviations

SPECT: Single Photon Emission Computed Tomography
PET: Positron Emission Tomography
R&D: Research and development
IAEA: International Atomic Energy Agency
EOB: End Of Bombardment
DOTA-NOC: DOTA (Ga)-D-Phe-Cys-1-Nal-D-Trp-Lys-Thr-Cys-Thr (ol), cyclic disulfide
DOTA-TATE: Gallate(1-), [N-[[4,7,10-tris[(carboxy-κ O)methyl]-1,4,7,10-tetraazacyclododec-1-yl-κ N1,κ N4,κ N7,κ N10]acetyl-κ O]-D-phenylalanyl-L-cysteinyl-L-tyrosyl-D-tryptophyl-L-lysyl-L-threonyl-L-cysteinyl-L-threonine cyclic disulfidato(4-)]-
PSMA-11: 4,6,12,19-Tetraazadocosane-1,3,7-tricarboxylic acid, 22-[3-[[[2-[[[5-(2-carboxyethyl)-2-hydroxyphenyl]methyl](carboxymethyl)amino]ethyl](carboxymethyl)amino]methyl]-4-hydroxyphenyl]-5,13,20-trioxo-, (3S,7S)-
PSMA-617: (3S,10S,14S)-3-[(naphthalen-2-yl)methyl]-1,4,12-trioxo-1-[(1r,4S)-4-[[2-[4,7,10-tris (carboxymethyl)-1,4,7,10-tetraazacyclododecan-1-yl]acetamido]methyl]cyclo-hexyl]-2,5,11,13-tetraazahexadecane-10,14,16-tricarboxylic acid
HBED: 3-(3-{[(2-{[5-(2-tert-Butoxycarbonyl-ethyl)-2-hydroxy-benzyl]-tert-butoxycarbonylmethyl-amino}-ethyl)-tert-butoxycarbonylmethyl-amino]-methyl}-4-hydroxy-phenyl)-propionic acid
GMP: Good Manufacturing Practice
ATSM: Hydrazinecarbothioamide, 2,2\cr’-(1,2-dimethyl-1,2-ethanediylidene) bis [N-methyl-
